# Dexmedetomidine for Managing Delirium and Agitation in Patients Admitted to Intensive Care Units: A Bibliometric Analysis

**DOI:** 10.7759/cureus.96219

**Published:** 2025-11-06

**Authors:** Nadia Aldhab Albadi, Omer Fathi Hassan Madani, Salim Marhoun Alshukaili, Sharifa Ali AlRawahi, Abhijit Nair

**Affiliations:** 1 Critical Care Medicine, Ibri Hospital, Ibri, OMN; 2 Anesthesiology, Ibra Hospital, Ibra, OMN; 3 Anesthesiology, Ibri Hospital, Ibri, OMN; 4 Anesthesiology, Nizwa Hospital, Nizwa, OMN

**Keywords:** bibliometric analysis, citespace, delirium in icu, dexmedetomidine, intensive care unit, vosviewer

## Abstract

Delirium affects almost one-third of patients admitted to the intensive care unit (ICU) and is responsible for increased mortality, prolonged mechanical ventilation, and long-term cognitive dysfunction. Dexmedetomidine, a selective α₂-adrenoceptor agonist, offers sedation resembling natural sleep, provides opioid-sparing analgesia, and, unlike opioids, causes minimal respiratory depression. However, it carries risks like bradycardia and hypotension in sick patients. We performed a comprehensive search of the Scopus database from January 2015 to August 2025 and identified 120 relevant articles after applying a predefined inclusion criterion. We used validated software (VOS viewer and CiteSpace) to find out various bibliometric details like co-authorship, co-occurrence, citation, bibliographic coupling, and co-citation. The results of our analysis emphasize the evolving prominence of dexmedetomidine in ICU management of delirium and agitation. Strengths of this study include using two tools for analysis, leading to a robust network visualization. However, the limitations involve relying only on the Scopus database and a time frame of 10 years. This study fills an important research gap and suggests future collaborations and research priorities, advocating for well-designed trials to address gaps in safe and effective use of dexmedetomidine.

## Introduction and background

Delirium affects up to one-third of intensive care unit (ICU) patients (the incidence varies from 2% to 80%) and is associated with higher mortality, longer ventilation times, and cognitive deficits. Dexmedetomidine is a highly selective α₂-adrenoceptor agonist that induces sedation resembling natural sleep, provides opioid-sparing analgesia and anxiolysis, and does not predispose to respiratory depression [1-3]. Dexmedetomidine is generally well tolerated, with minimal respiratory depression. However, cardiovascular effects such as bradycardia and hypotension are notable [4].

The results of the Sedation Practice in Intensive Care Evaluation III (SPICE III) trial mentioned that a higher dose of dexmedetomidine is required to reach sedation targets, thereby increasing the incidence of bradycardia and hypotension [5]. However, the authors observed that the overall mortality difference at 90 days was the same. A randomized study involving 74 ICU patients concluded that the addition of dexmedetomidine to mechanical ventilation in the presence of agitated delirium resulted in more ventilator-free hours at seven days than the standard (placebo) [6].

A systematic review, meta-analysis, and trial sequential analysis published by Ng et al involving 25 studies (3,240 patients) concluded that the use of dexmedetomidine reduced the incidence of delirium and agitation in intensive care patients when compared to placebo or standard of care, with a level of evidence being moderate to high [7]. Wang et al. published a meta-analysis including 36 studies (9,623 patients in total) to compare delirium risk in ICU patients with or without dexmedetomidine [8]. The authors concluded that dexmedetomidine was linked to a clinically insignificant decrease in delirium risk, length of ICU/hospital stay, and duration of mechanical ventilation, according to low- or very low-quality evidence. However, it was not linked with reduced mortality or shorter delirium duration in ICU patients.

Although there are several clinical trials, review articles including systematic review and meta-analysis published to investigate dexmedetomidine in managing ICU delirium and agitation, a bibliometric analysis is missing, considering the ease of availability, popularity, and reasonable safety of the medication. The aim of performing this bibliometric analysis is to add a complementary, quantitative layer to traditional evidence synthesis by mapping how research on dexmedetomidine for ICU delirium and agitation has developed, which work drives the field, what topics and article categories dominate, and what critical gaps remain, thereby guiding further trials in the future, facilitating collaborations, and implementation efforts.

## Review

Methods

From January 2015 through August 2025, we conducted a literature search. To reduce bias from database updates, all literature searches and data downloads were all done on one day (August 15, 2025). We conducted a comprehensive search on the Scopus database using the following search strategy: [Title] OR [Abstract] OR [Keyword]= (TITLE-ABS-KEY (dexmedetomidine) AND TITLE-ABS-KEY (delirium) OR TITLE-ABS-KEY (agitation AND TITLE-ABS-KEY (intensive care unit) AND NOT TITLE-ABS-KEY (postoperative) ) AND PUBYEAR > 2015 AND PUBYEAR < 2026 AND ( EXCLUDE ( SUBJAREA , "IMMU" ) OR EXCLUDE ( SUBJAREA , "DENT" ) ) AND ( LIMIT-TO ( LANGUAGE , "English" ) ). The Scopus file was saved in a comma-separated values (CSV) format.

Complete records and cited references were included in the exported data, which were utilized for analysis. The bibliometric analysis was conducted using VOSviewer (Version 1.6.20; Leiden University, Leiden, the Netherlands). Using the software, we performed five distinct kinds of analyses: bibliographic coupling, co-authorship, co-occurrence, citation, and co-citation. For co-authorship analysis, the units of analysis were authors, organizations, and countries. For co-occurrence analysis, the units of analysis included all keywords, author keywords, and index keywords. In citation and bibliographic coupling, the units of analysis were documents, sources, authors, organizations, and countries. For co-citation, the units of analysis were cited references, cited sources, and cited authors [9]. The CSV file was then uploaded to the VOSviewer software for bibliometric analysis in various categories.

Additionally, we utilized the CiteSpace tool (version 6.3, created by Dr. Chaomei Chen, a professor in the College of Computing and Informatics at Drexel University, Philadelphia, Pennsylvania, USA), a Java-based program, which generates cluster timelines, co-citation networks, and keyword co-occurrence maps. The tool provides visual outputs for co‑authorship networks, co‑citation clusters, and keyword burst timelines to draw attention to new lines of inquiry and conceptual frameworks. CiteSpace's built-in conversion tool, which allows the breakdown of bibliographic records, was used to transform the CSV file into a Web of Science-compatible format for CiteSpace analysis [10].

Results

From the 638 documents that were available from the initial Scopus database search, 120 articles fulfilled the inclusion criteria. The CSV file was then analyzed using VOSviewer and CiteSpace software. Table 1 provides a summary of the total number of articles in various categories.

**Table 1:** Summary of various types of articles

Type of article	Number of articles (n=120)
Original articles	
Randomized controlled trials	18
Research articles (observational, descriptive, clinical study, comparative/non-randomized, survey)	21
Retrospective studies	9
Review articles	
Narrative reviews	34
Systematic review and/or meta-analysis	13
Umbrella review	2
Case report/series	14
Miscellaneous	
Editorial	1
Letter to Editor/comment	3
Study protocol	5

Co-authorship

**Figure 1:**
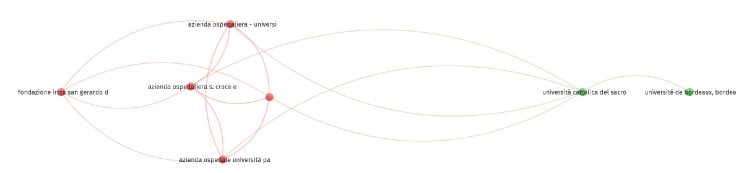
Co-authorship and organization network diagram IRCCS: Istituto di Ricovero e Cura a Carattere Scientifico.

Co-authorship analysis examines collaborative patterns among contributors to scientific publications. It identifies and visualizes the relationships between authors, institutions, and countries based on shared authorship of scholarly works. Out of the 663 authors, three met the threshold for co-authorship and authors analysis. The three authors are Lisa Burry, Linda I. Chlan, and Michael C. Reade. All authors have three documents each, but Michael C. Reade has 310 citations for three documents, which is the maximum. Out of the 432 organizations, 21 met the threshold. We created a network with all 21 organizations, out of which seven were connected (Figure 1). The top 10 organizations are summarized in Table 2. All 10 organizations have two documents each, but the highest number of citations was from Fondazione Istituto di Ricovero e Cura a Carattere Scientifico (IRCCS) San Gerardo dei Tintori, Monza, Italy (257 citations). Out of the 33 countries, eight met the threshold. The USA had 48 documents and a corresponding 1,539 citations, which was the highest of all (Table 2).

**Table 2:** Table summarizing co-authorship: organizations and countries IRCCS: Istituto di Ricovero e Cura a Carattere Scientifico, VA: Veteran Affairs.

Co-authorship and organizations		
Organization	Documents	Citations
Azienda Ospedale Università Padova, Padova, Italy	2	38
Fondazione IRCCS San Gerardo dei Tintori, Monza, Italy	2	257
IRCCS Azienda Ospedaliero-Universitaria di Bologna, Bologna, Italy	2	38
University of Ottawa, Canada	2	24
Università Cattolica del Sacro Cuore, campus di Roma, Rome, Italy	2	43
VA Medical Centre, United States	2	172
Vanderbilt University Medical Center, Nashville, United States	2	145
Yale New Haven Hospital, New Haven, United States	2	129
Co-authorship and countries		
Country		
Australia	7	404
Canada	14	820
China	24	535
France	9	309
Germany	5	242
Italy	8	446
United Kingdom	9	457
United States	48	1,539

Co-occurrence

Co-occurrence analysis is a bibliometric technique that looks at the frequency and patterns of specific terms (usually keywords) appearing together in scientific publications in order to investigate the conceptual structure of a research domain. Within a field, this approach aids in identifying thematic clusters, new trends, and intellectual connections.

**Figure 2:**
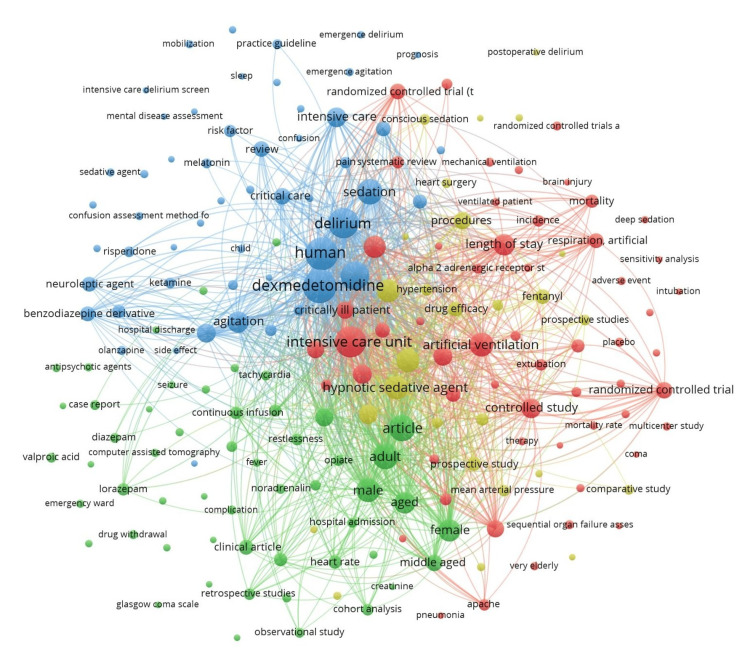
Co-occurrence and all keywords

**Figure 3:**
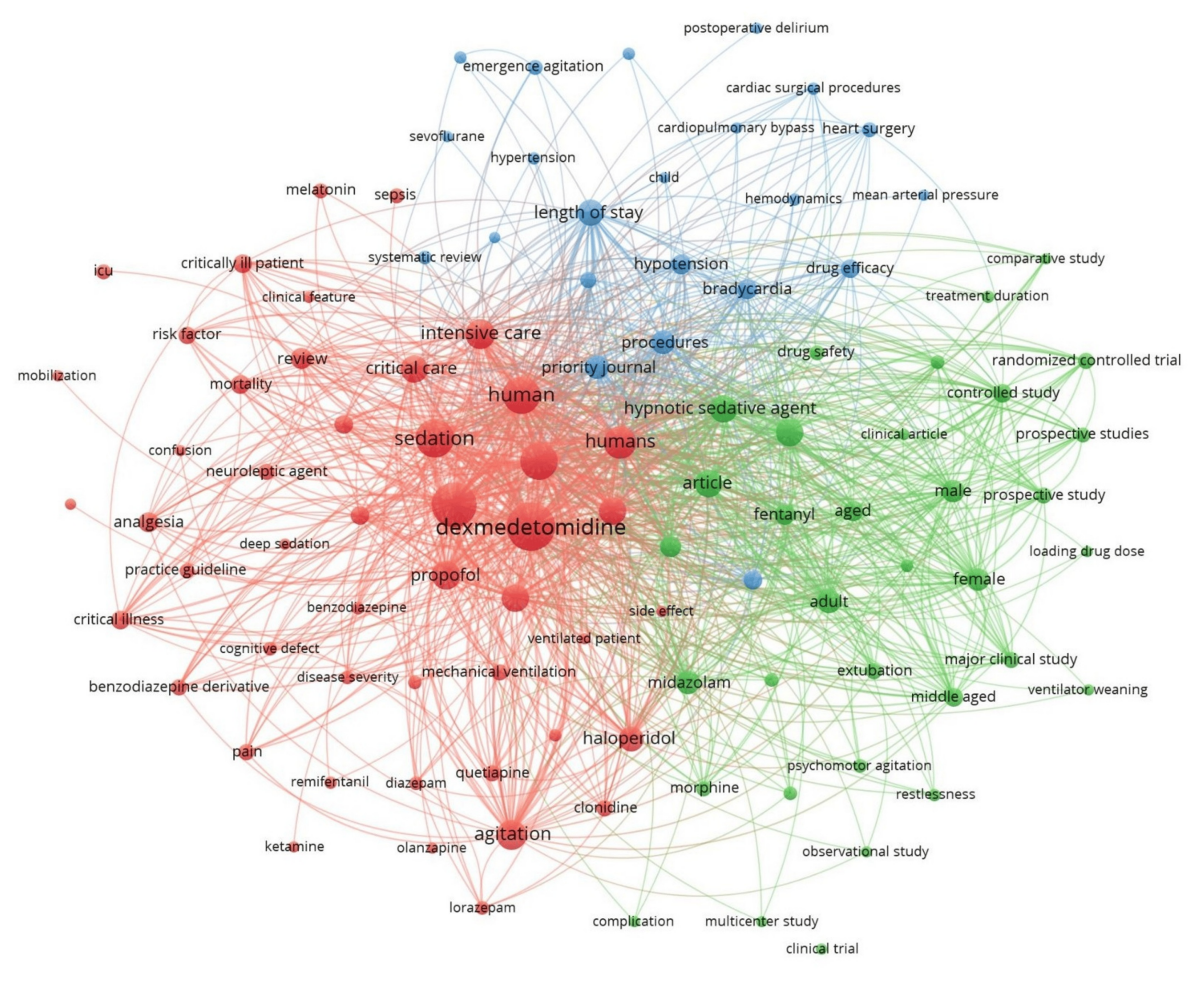
Co-occurrence and author keywords

**Figure 4:**
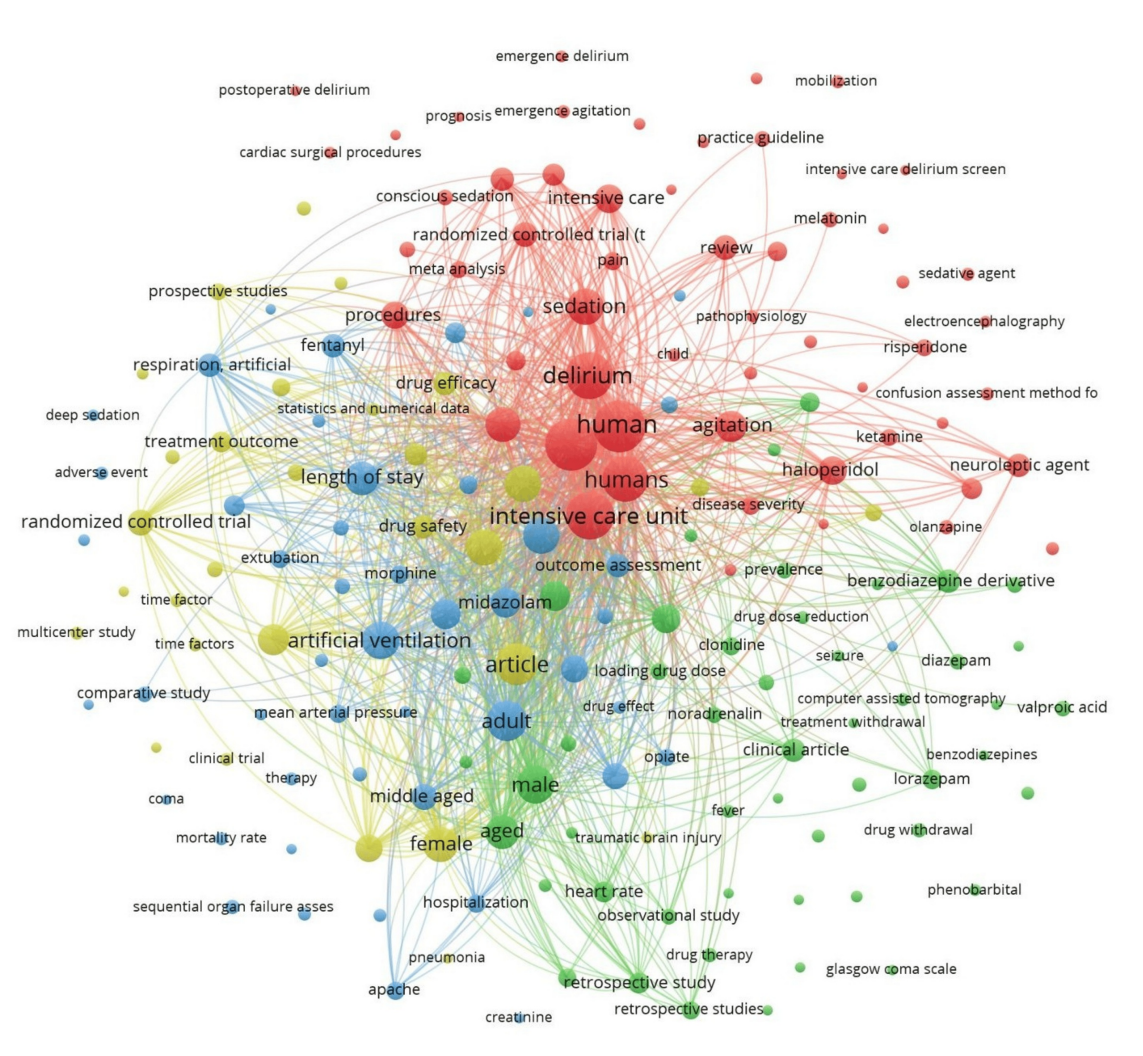
Co-occurrence and index keywords

Of the 1,441 keywords, 191 met the threshold for a minimum of five occurrences of a keyword. We created a network using all 191 keywords (Figure 2). The keyword ‘dexmedetomidine’ had a total of 116 occurrences. Of the 901 author keywords, 104 met the threshold. We created a network using all 104 author keywords (Figure 3) and summarized the top 10 author keywords in Table 3. The keyword ‘dexmedetomidine’ had a total of 85 occurrences. Of the 1,315 index keywords, 184 met the threshold. We created a network using all the 184 author keywords (Figure 4) and summarized the top 10 author keywords in Table 3. The keyword ‘human’ had 113 occurrences, and ‘dexmedetomidine’ had a total of 109 occurrences.

**Table 3:** Table summarizing co-occurrence: author keywords and index keywords

Co-occurrence and author keywords	
Keyword	Occurrences
article	27
artificial ventilation	28
delirium	70
dexmedetomidine	85
human	49
humans	37
hypnotic sedative agent	28
hypnotics and sedatives	28
intensive care unit	49
sedation	52
Co-occurrence and index keywords	
keyword	
adult	65
article	72
artificial ventilation	59
delirium	88
dexmedetomidine	109
human	113
humans	84
hypnotics and sedatives	55
intensive care unit	96
male	59

Citation Analysis

A bibliometric technique for assessing the intellectual influence, scholarly impact, and dissemination of scientific publications is citation analysis. It involves quantifying and mapping citation relationships among documents, authors, journals, institutions, and countries to understand how knowledge flows and accumulates within a research domain. Of the 120 documents, 83 met the threshold. We summarized the top 10 documents in Table 4.

**Table 4:** Citation analysis and documents

Document	Citations	Links
Wu et al. (2022) [11]	2	2
Thomas et al. (2021) [12]	25	1
Wang et al. (2021) [8]	20	3
Qiu et al. (2020) [13]	19	1
Wu et al. (2018) [14]	84	2
Ganau et al. (2018) [15]	53	1
Romagnoli et al. (2018) [16]	29	2
Carrasco et al. (2016) [17]	85	1
Lu et al. (2016) [18]	8	1
Porhomayon et al. (2016) [19]	37	0

**Figure 5:**
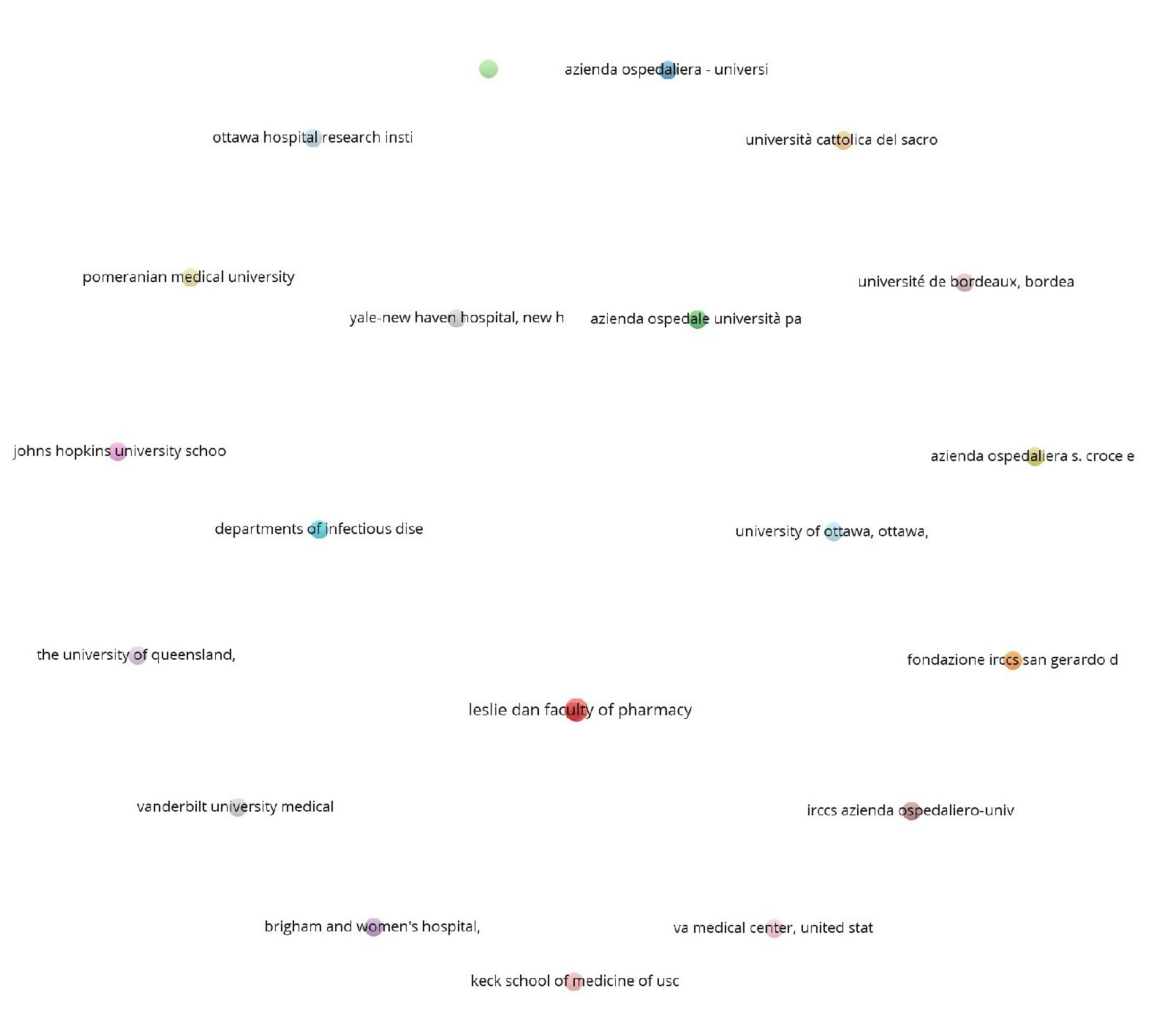
Network diagram depicting citation analysis for organizations

The document by Carrasco et al. had a maximum of 85 citations. Of the 81 sources, eight met the threshold. Out of these eight sources, only three were connected. Among eight sources, JAMA had 958 citations for three documents, which was the maximum. The eight sources are summarized in Table 5. Out of 663 authors, three met the threshold. Author Michael C. Reade had 310 citations for three documents authored. Of the 432 organizations, 21 met the threshold. We created a network using all 21 organizations. The leading organization was Brigham and Women's Hospital from Boston, United States, with 707 citations for two documents (Figure 5). We summarized the 21 organizations in Table 6. Of the 33 countries, eight met the threshold. The United States had the maximum number of documents, 48, with a maximum of 1,539 citations (Table 7).

**Table 5:** Citation analysis and sources JAMA: Journal of American Medical Association, BMC: Biomed Central.

Source	Documents	Citations
BMC Anesthesiology	6	49
Critical Care Medicine	3	106
JAMA	3	958
Journal of Clinical Anesthesia	4	108
Journal of Intensive Care Medicine	3	19
Minerva Anestesiologica	3	85
Neurocritical Care	3	43
Trials	3	18

**Table 6:** Citation analysis and organizations IRCCS: Istituto di Ricovero e Cura a Carattere Scientifico, USC: University of South California.

Organization	Documents	Citations
Azienda Ospedale Università Padova, Padova, Italy	2	38
Azienda Ospedaliero-Universitaria Città Della Salute e Della Scienza di Torino, Turin, Italy	2	38
Azienda Ospedaliera Santa Croce e Carle, Cuneo, Italy	2	38
Brigham and Women's Hospital, Boston, United States	2	707
Department of Infectious Diseases, Karolinska University Hospital, Solna, Sweden	2	21
Fondazione IRCCS San Gerardo dei Tintori, Monza, Italy	2	257
IRCCS Azienda Ospedaliero-Universitaria di Bologna, Bologna, Italy	2	38
Johns Hopkins University School of Medicine, Baltimore, United States	2	661
Keck School of Medicine of USC, Los Angeles, United States	2	59
Leslie Dan Faculty of Pharmacy, University of Toronto, Toronto, Canada	3	14
Mayo Clinic Scottsdale-Phoenix, Scottsdale, United States	2	12
Ottawa Hospital Research Institute, Ottawa, Canada	2	24
Pomeranian Medical University in Szczecin, Poland	2	179
The University of Queensland, Brisbane, Australia	2	294
University of Ottawa, Ottawa, Canada	2	24

**Table 7:** Citation analysis and countries

Country	Documents	Citations
Australia	7	404
Canada	14	820
China	24	535
France	9	309
Germany	5	242
Italy	8	446
United Kingdom	9	457
United States	48	1,539

Bibliographic Coupling

Bibliographic coupling is a bibliometric technique used to assess intellectual similarity between entities like documents, authors, institutions, sources, and countries based on shared references. When any two entities cite one or more common sources, they are known as bibliographically coupled. This method helps identify thematic proximity, research alignment, and knowledge structures within a scientific domain.

**Figure 6:**
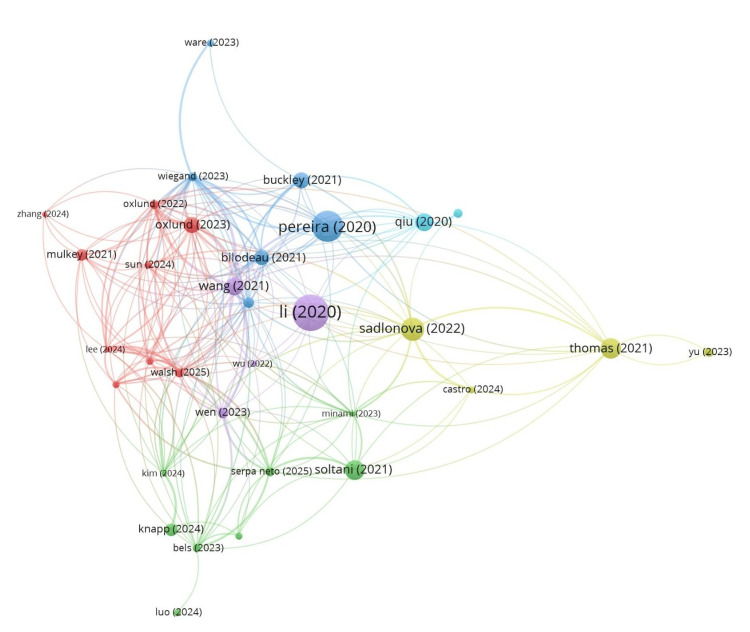
Bibliographic coupling and all 83 documents

**Figure 7:**
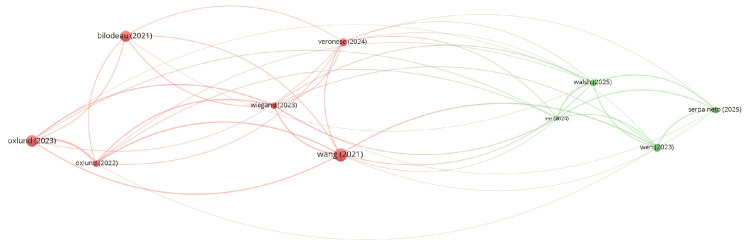
Bibliographic coupling and top 10 documents

**Figure 8:**
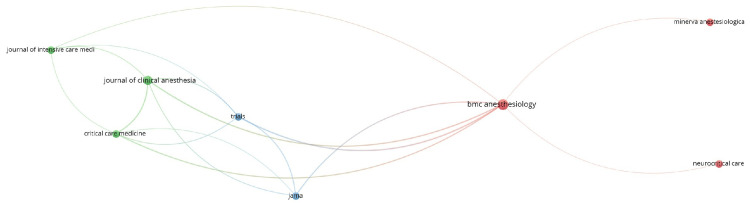
Bibliographic coupling and sources

**Figure 9:**
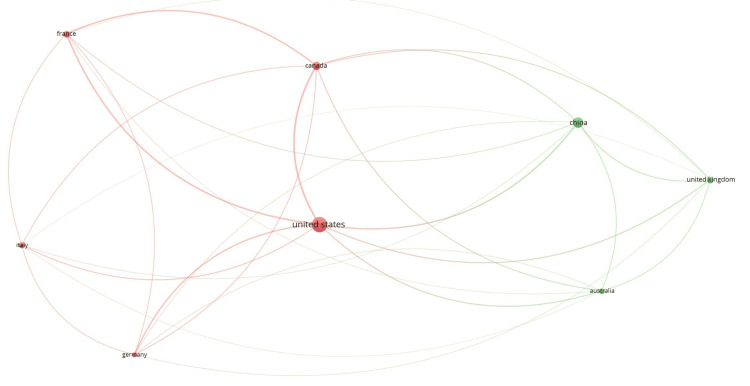
Bibliographic coupling and countries

Of the 120 documents, 83 met the threshold. We created a network of all 83 documents (Figure 6) and also with the top 10 documents (Figure 7). The summary of the top 10 documents based on the total link strength is presented in Table 8. The document by Wang et al. (2021) [8] had the maximum of 20 citations and the highest link strength of 52. Out of 81 sources, eight met the threshold. We created a network using all eight sources (Figure 8) and summarized them in Table 9. Among eight sources, JAMA had 958 citations for three documents. Out of 663 authors, three met the threshold. Author Michael C. Reade had 310 citations for three documents authored. Of the 432 organizations, 21 met the threshold. The University of Queensland, Brisbane, Australia, had the maximum number of citations, with 294 for two documents. Table 9 provides a summary of the top 10 organizations with bibliographic coupling. Out of 33 countries, eight met the threshold. We created a network using all eight countries (Figure 9) and summarized it in Table 9. The USA had the maximum citations of 1,539 for 48 documents.

**Table 8:** Summary of top 10 documents for bibliographic coupling

Document	Citations	Total link strength
Walsh et al. (2025) [20]	5	32
Lee et al. (2024) [21]	2	33
Serpa Neto et al. (2025) [22]	5	19
Veronese et al. (2024) [23]	7	24
Wiegand et al. (2023) [24]	5	49
Oxlund et al. (2023) [25]	16	50
Wen et al. (2023) [26]	7	28
Oxlund et al. (2022) [27]	5	56
Bilodeau et al. (2021) [28]	14	21
Wang et al. (2021) [8]	20	52

**Table 9:** Bibliographic coupling summary for sources, organizations, and countries

Bibliographic coupling and sources		
Source	Documents	Citations
BMC Anesthesiology	6	49
Critical Care Medicine	3	106
JAMA	3	958
Journal of Clinical Anesthesia	4	108
Journal of Intensive Care Medicine	3	19
Minerva Anestesiologica	3	85
Neurocritical Care	3	43
Trials	3	18
Bibliographic coupling and organizations		
Organization		
Departments of Infectious Diseases, Karolinska University Hospital, Solna, Sweden	2	21
Leslie dan Faculty of Pharmacy, University of Toronto, Canada	3	14
The University of Queensland, Brisbane, Australia	2	294
University of Ottawa, Canada	2	24
University of Toronto, Canada	2	135
Università Cattolica del Sacro Cuore, Campus di Roma, Italy	2	43
Université de Bordeaux, Bordeaux, France	2	17
VA Medical Centre, United States	2	172
Vanderbilt University Medical Center, Nashville, United States	2	145
Yale New Haven Hospital, New Haven, United States	2	129
Bibliographic coupling and countries		
Country	Documents	Citations
Australia	7	404
Canada	14	820
China	24	535
France	9	309
Germany	5	242
Italy	8	446
United Kingdom	9	457
United States	48	1,539

Co-citation

Co-citation analysis analyzes how frequently two entities like references, authors, or sources are cited together in other publications. When two entities are co-cited by a third, it is suggestive of a thematic relationship between them. This helps identify foundational literature, influential scholars, and thematic clusters within a domain.

**Figure 10:**
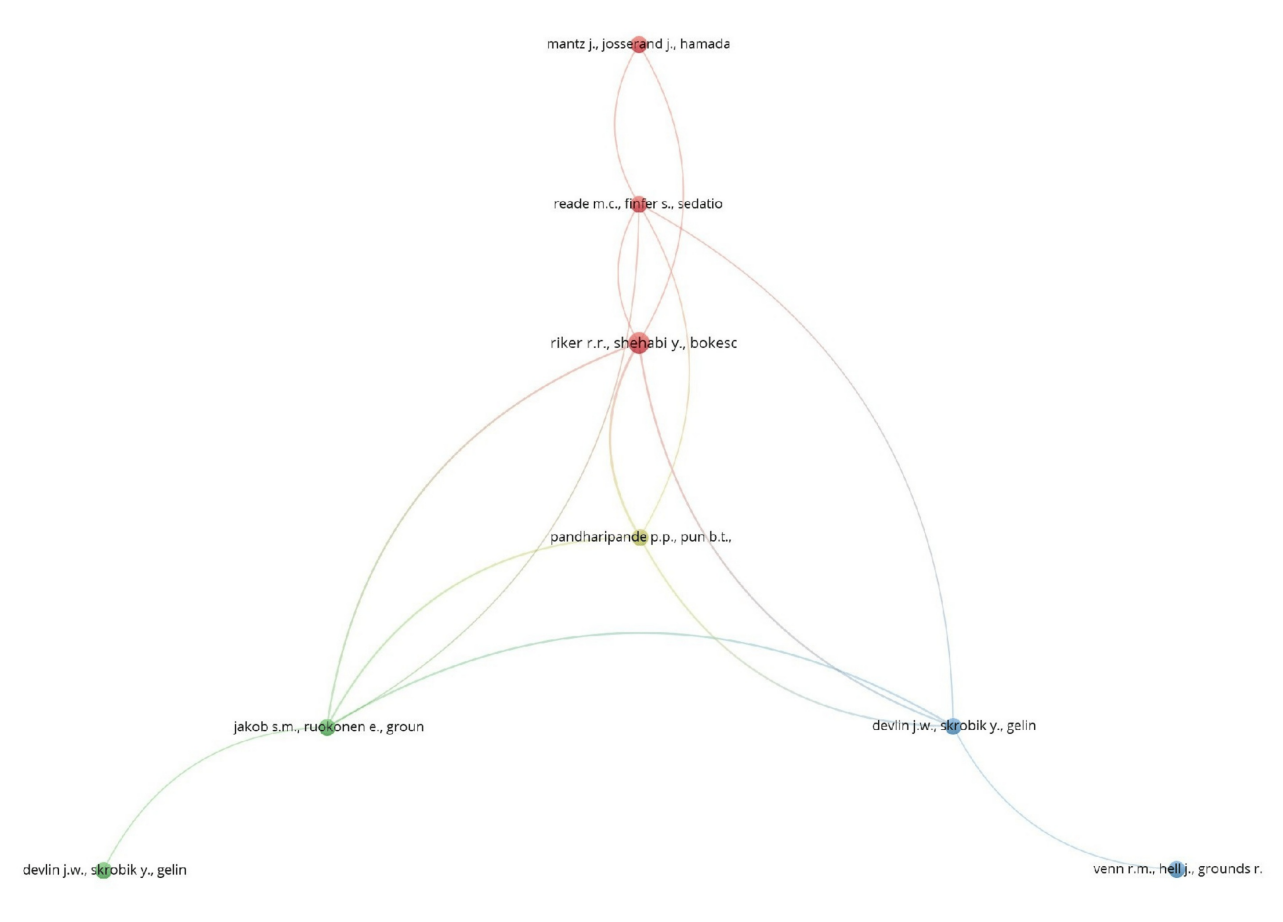
Co-citation analysis and cited references

**Figure 11:**
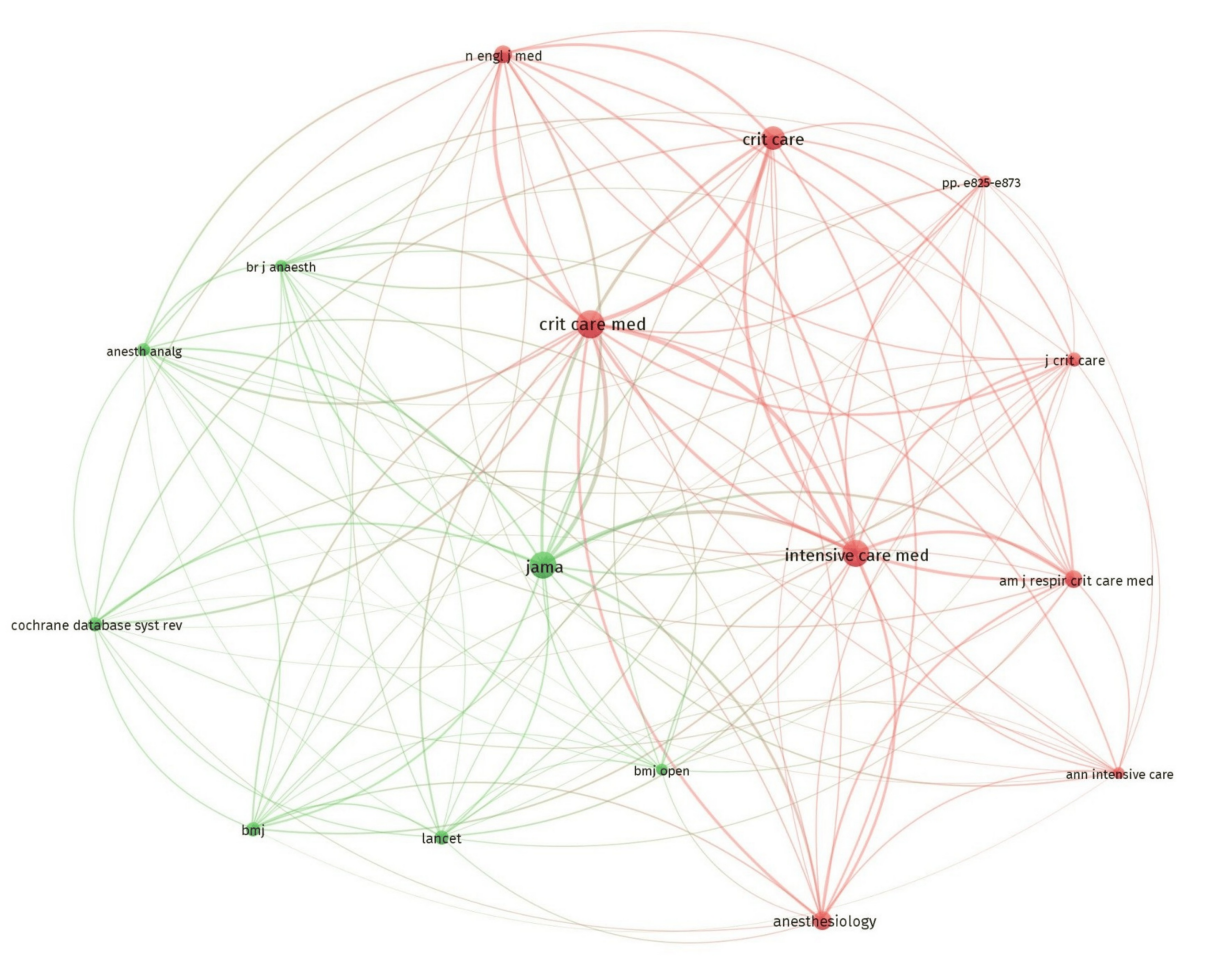
Co-citation analysis and cited sources

**Figure 12:**
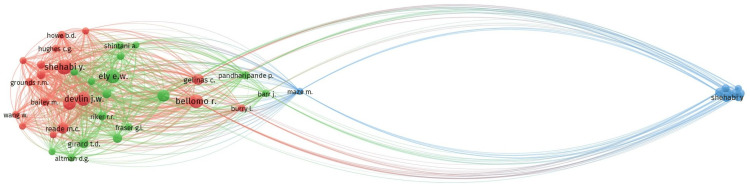
Co-citation analysis and cited authors

Of the 2,283 references, 11 met the threshold, considering a minimum of three citations for each cited reference. We created a network with all 11 references (Figure 10) and summarized the details in Table 10. The article by Riker et al. [29] had a maximum of five citations. Out of 925 cited sources, 16 met the threshold, considering 20 minimum citations from a source. We created a network using all 16 sources (Figure 11) and summarized the top 10 sources in Table 11. The journal Critical Care Medicine had the highest citations at 105. Out of 6,342 authors, 48 met the threshold, considering a minimum of 10 citations of an author. We created a network initially using all 48 authors (Figure 12) and then summarized the top 10 authors in Table 11. The author Y. Shehabi had the maximum of 49 citations among authors.

**Table 10:** Summary of co-citation analysis and cited references

Cited reference	Citations
Devlin et al. [30]	3
Jakob et al. [31]	3
Keating [32]	3
Lewis et al. [33]	3
Mantz et al. [34]	3
Pandharipande et al. [35]	3
Reade and Finfer [36]	3
Reade et al. [37]	3
Riker et al. [29]	5
Venn et al. [38]	3

**Table 11:** Co-citation analysis for cited sources and cited authors

Co-citation and cited sources	
Source	Citations
Am J Respir Crit Care Med	44
Anesth Analg	25
Anesthesiology	46
Br J Anaesth	21
Crit Care	75
Crit Care Med	105
Intensive Care Med	95
J Crit Care	26
JAMA	93
N Engl J Med	42
Co-citation and cited authors	
Author	
Bailey M	15
Bellomo R	41
Devlin JW	40
Ely EW	41
Gelinas C	26
Pandharipande PP	35
Reade MC	25
Ruokonen E	15
Shehabi Y	49
Skrobik Y	31

Analysis Using CiteSpace Software

**Figure 13:**
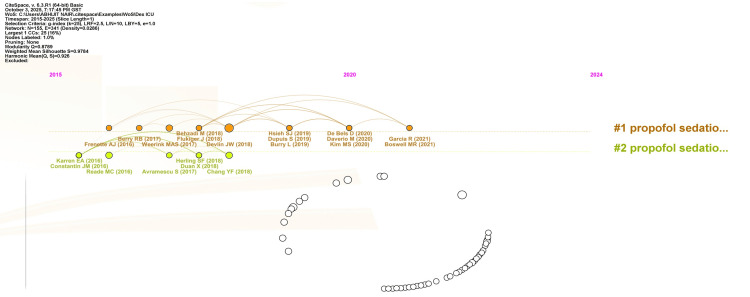
Figure depicting a document co-citation analysis network generated using CiteSpace, delineating the intellectual landscape and thematic evolution of research on the use of dexmedetomidine for the management of delirium and agitation in adult ICU patients from 2015 to 2025 LRF: log-likelihood ratio frequency, L/N: links per node, LBY: label by year, CC: cluster centrality (or citation count).

As analyzed by CiteSpace, Figure 13 shows the document co-citation network for publications discussing the use of dexmedetomidine in the treatment of agitation and delirium in adult ICU patients. The two primary clusters, which are indicated by the color's orange and light green, represent the changing concepts and areas of study surrounding the clinical use of dexmedetomidine. Crucial studies that reflect both foundational advancements and the interconnectedness of emerging insights are those by Williamson et al. (2016) [39] and Devlin et al. (2018) [30]. The analysis highlights a strong and well-structured body of literature, with high modularity and silhouette scores that point to developed, discrete research areas in this area.

**Figure 14:**
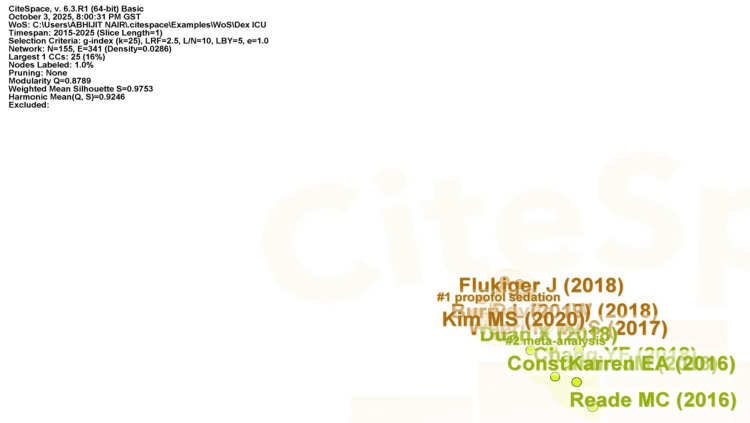
Document co-citation analysis network generated by CiteSpace for publications concerning dexmedetomidine in the management of delirium and agitation in adult ICU patients (2015-2025) LRF: log-likelihood ratio frequency, L/N: links per node, LBY: label by year, CC: cluster centrality (or citation count).

Figure 14 depicts a document co-citation analysis network focused on the research landscape of dexmedetomidine in managing delirium and agitation among adult ICU patients within the period 2015-2025. The visualization highlights two distinct thematic clusters. Cluster #1, in orange, is for “propofol sedation". This group consists of articles primarily examining the clinical use of dexmedetomidine in the context of propofol-based sedation strategies and comparative efficacy in adult ICU patients. Influential contributions within this cluster include Flukiger et al. (2018) [40], Kim et al. (2020) [41], Dupuis et al. (2019) [42], and Duan et al. (2018) [43], suggesting that these works underpin much of the contemporary discourse surrounding sedation modalities and their clinical impact. Cluster #2, which is in green, depicts "meta-analysis". This cluster displays systematic reviews and meta-analyses, such as those by Reade et al. (2016) [6], Constantin et al. (2016) [44], and Karren et al. (2016) [45], which synthesize existing evidence and provide quantitative insights into delirium and agitation management in the ICU setting. High silhouette (S=0.9753) and modularity (Q=0.8708) scores show the existence of distinct and cohesive clusters, indicating maturity and structure in this field of study. The visualization's lack of extensive links or extra clusters emphasizes the current research's narrow focus and concentrated intellectual foundation.

In the figures, Q represents the journal’s quartile within its Scopus subject category, showing its rank band among journals in that field. Quartiles run from Q1 (top 25%) to Q4 (bottom 25%) and are derived from the journal’s rank in the category. S represents the journal’s percentile score (the Scopus percentile), a number from 0 to 100 that shows the journal’s relative position within its category based on the CiteScore metric. A higher S means the journal is ranked closer to the top of its category.

Discussion

This bibliometric analysis provides a comprehensive overview of the research landscape on dexmedetomidine for managing delirium and agitation in ICU patients from 2015 to 2025, highlighting key contributors, thematic clusters, and influential works. This is likely the first bibliometric analysis to map the literature on dexmedetomidine use in managing ICU delirium and agitation.

In addition to mapping publication trends, this analysis reflects the changing philosophy in critical care, which is moving away from immobilization and sedation based on opioids and benzodiazepines and toward neuroprotection and humane, physiological sedation. Multidisciplinary research (critical care, anesthesiology, neuropsychology, and pharmacology) has come together around dexmedetomidine as a sedative for delirium prevention and management, as highlighted by the collaboration networks and citation clusters. Mortality benefits are still being demonstrated with the use of dexmedetomidine, even with a lower incidence of delirium [33,36]. Additionally, hemodynamic instability and cost concerns prevent its widespread use, especially in resource-constrained ICUs. There is a lot of scope for well-designed trials that could investigate dose optimization, sedation depth titration, and long-term cognitive impairments following ICU discharge. Previously, Ma et al. published a bibliometric analysis and attempted to synthesize the progress in understanding ICU delirium [46]. They broadly grouped ICU delirium into several categories, including various risk factors, clinical presentation, diagnosis, and modalities of prevention and treatment, utilizing emerging keywords and literature clustering. Our analysis is more focused in its approach and investigates the use of one medication in ICU delirium and agitation.

The dominance of the United States in co-authorship and citations, evident from 48 documents and 1,539 citations, emphasizes its leading role in advancing critical care research in the form of well-designed clinical trials. The analysis identifies influential authors like Michael C. Reade (three documents, 310 citations) and organizations like Brigham and Women's Hospital (two documents, 707 citations). These findings reveal the expertise of these authors and organizations in managing delirium and agitation, practicing various sedation strategies, and thereby making global efforts to optimize ICU outcomes. Citation and bibliographic coupling analyses revealed important papers, like the one by Wang et al. (2021) [8], with a high link strength of 52. This meta-analysis evaluated the modest but clinically significant advantages of dexmedetomidine in lowering the incidence of delirium and the length of ventilation. Co-citation trends brought to light important sources such as Riker et al. (2009) [29], which influenced later RCTs by comparisons with other sedatives like midazolam.

The bibliometric landscape of dexmedetomidine research in ICU delirium and agitation reveals a robust pattern of collaborative scholarship, mainly in North America, Europe, and parts of Asia, with important authors like Michael C. Reade and institutions like Brigham and Women's Hospital. MC Reade is at the forefront of influential networks. As demonstrated by the division into clusters for propofol-comparative sedation research and meta-analytical syntheses, the thematic evolution also reflects maturation from basic clinical efficacy and safety studies into more complex inquiries addressing long-term cognitive outcomes and cost-effectiveness. In order to improve delirium management procedures in the ICU, this trajectory highlights a dynamic and increasingly complex research environment that is ready to incorporate multidisciplinary viewpoints and cutting-edge analytics.

This bibliometric analysis has several limitations. We utilized only one database, i.e., Scopus, thereby possibly missing articles not indexed in Scopus and also other non-indexed publications. We included articles involving adult patients only, thereby further narrowing our search, but at the same time, we were more specific. We analyzed the articles for over a decade, from 2015 to 2025, thus capturing recent trends but omitting earlier foundational work. The major strength lies in using two validated tools for analysis (VOSviewer and CiteSpace), enabling multifaceted insights into networks and bursts. Future research should prioritize multicenter studies in diverse populations and integrate AI-driven dosing models to enhance precision, addressing gaps in long-term cognitive outcomes.

## Conclusions

This bibliometric analysis thoroughly summarizes the important research over the last 10 years about the use of dexmedetomidine to treat agitation and delirium in ICU patients. The widespread use of dexmedetomidine in the practice of critical care is demonstrated by the predominance of highly influential organizations and authors from the US, Europe, and Asia. In order to uncover mature and unique research clusters focused on sedation depth, delirium prevention, and comparative sedative efficacy, co-citation and bibliographic coupling analyses were used to identify important works and journals that influenced the field. Future well-designed and collaborative research could bridge existing gaps by optimizing dosing strategies, duration, evaluating cost-effectiveness, and standardizing delirium assessment methodologies to enhance patient outcomes in the ICU.
